# Dietary Diversity and Its Related Factors among Adolescents: A Survey in Ahvaz-Iran

**DOI:** 10.5539/gjhs.v5n2p181

**Published:** 2012-01-13

**Authors:** Mahdis Vakili, Parvin Abedi, Mehrdad Sharifi, Mostafa Hosseini

**Affiliations:** 1 Department of Nutrition, Para Medicine School, Member of Student Research Center of Ahvaz Jundishapur Medical University of Sciences, Ahvaz, Iran; 2 Department of Midwifery, Reproductive Health Research Center, Ahvaz Jundishapur University of Medical Sciences, Ahvaz, Iran; 3 Director of the East Health Centre in Ahvaz, Ahvaz Jundishapur University of Medical Sciences, Ahvaz, Iran; 4 Department of Epidemiology and Biostatistics, School of Public Health, Tehran University of Medical Sciences, Tehran, Iran

**Keywords:** dietary diversity, adolescents’ girls, high school, economical situation, FAO's guideline

## Abstract

**Introduction:**

Healthy growth and development essentially need a balanced diet of nutrients and vitamins which includes a variety of foods from different food groups. The primary aim of this study was to assess the dietary diversity (DD) and its related factors among adolescents’ high school girls in Ahvaz-Iran.

**Methodology:**

This was a cross-sectional study which it was structured based on the WHO & FAO's dietary diversity questionnaire. The study population consisted of 506 high school girls aged 15 to 18. Data about diet, socio-demographic and anthropometric characteristics were gathered. A dietary diversity score (DDS) and anthropometric of girls were measured. The relation of DDS with anthropometric measures and economic situation were assessed.

**Results:**

The mean DDS was 6.81±1.75. A total of 18.85% and 8.3% of participants were overweight and obese respectively. In participants with scores ≥ six, Body Mass Index, waist circumference and waist-hip ratio were slightly greater than in individuals with scores less than six, however it was not significant except for waist hip ratio. The Logistic Regression showed that; weak economical situation was a risk for poor DDS (OR= 3.5, CI= 1.06-10.6, p=0.03).

**Conclusion:**

The FAO's third version of guidelines is a good indicator for measuring DDS. The results of this study indicate that high school girls’ knowledge and practice about dietary diversity are not good and need to be improved by educational classes.

## 1. Introduction

Nutrition is a main component of health and development. Healthy eating is related to the infant evolution, maternal and child health, healthier pregnancy and delivery, lower risk of chronic diseases and better academic achievement (World Health Organization, 2011). Dietary diversity (DD) and the amount of animal source foods that an individual consumes are two commonly used measures for dietary quality. Healthy growth and development essentially need a balanced diet of nutrients and vitamins which includes a variety of foods from different food groups (vegetables, fruits, grains, and animal source foods) (Belachew et al., 2004).

With regard to dietary factors that are associated with increased risk of chronic diseases, nutritional advice promote dietary diversity and reduced intake of non-healthy food items such as fat, salt and refined sugars (Ruel, 2003a). Monotonous diets based on starchy staples lack essential micronutrients and contribute to the burden of malnutrition and micronutrient deficiencies. Food-based strategies are prepared to meet micronutrient needs as a first priority (Allen, 2008).

Dietary diversity can be measured at the household or individual level through use of a questionnaire. Most often it is measured by counting the number of food groups rather than the food items consumed. At the household level, dietary diversity is usually considered as a measure of access to food (e.g., of households’ capacity to access costly food groups); while at the individual level it reflects dietary quality, mainly the micronutrient adequacy of the diet. Although the reference period can vary, it is most often the previous day or week (Food and Agriculture Organization, 2011).

Dietary diversity instruments have recently become the preferred method for studying dietary adequacy in developing countries. These score points are based on different food items or food groups in a certain given period ranged from 1 to 15 days (Administrative Committee on Coordination/Subcommittee on Nutrition, 2005).

Few studies aimed to investigate the dietary diversity against adequacy of nutrient in developing countries, found a good relationship with studies in developed countries (Ruel, 2003b). The lack of dietary diversity is a severe problem among poor people in the developing countries suggests that they feed mostly on starchy staples without or with minimal use of animal products, fresh fruits and vegetables (Popkin, 1994). The FAO's third version of the guidelines for measuring household and individual dietary diversity which was revealed in August 2007 is introduced as an expedient instrument for DDS measures (Food Agriculture Organization, 2007).

Hoddinott and Yohannes (2002) studied the association between household dietary diversity scores (DDS) and dietary energy availability in ten countries. The study results suggest that the DDS has the potential for monitoring changes in dietary energy availability, particularly when resources are lacking quantitative measurements.

Recently the association between dietary diversity and micronutrient adequacy of diets of women in reproductive age was assessed in five countries. Dietary diversity was significantly associated with micronutrient adequacy in all sites (Arimond et al., 2010). Also the DDS was found to be a useful indicator of some specific nutrient adequacy in women from Tehran (Mirmiran, Azadbaht, & Azizi, 2006). The primary aim of this study was to evaluate dietary diversity and its related factors among adolescent high school girls in Ahvaz, Iran.

## 2. Materials and Methods

This was a cross-sectional study, which was structured based on the WHO & FAO's dietary diversity questionnaire that was revealed in 2003. Also, we used the FAO's third version of the guidelines for measuring household and individual dietary diversity which was revealed in August 2007 (Hoddinott & Yohannes, 2002).

### 2.1 Study Population

The study population comprised 534 high school girls aged 15 to 18 who were studying in the government and private high schools in Ahvaz, 2011. The study design was approved by the Ethics Committee of the Ahvaz Jundishapur University of Medical Sciences (project number: 89S.187). The sample size was determined by considering a confidence interval of 95% and was based on the statistics about high school student populations, which were obtained from the official Education Department of Ahvaz. We used cluster random sampling and the school population was organized according to the different areas and regions in Ahvaz and the type of schools, so that it could represent students throughout the city. Seventeen high schools were randomly selected from the four educational districts in Ahvaz. Then one class in each school was randomly selected. An informed written consent was obtained from the study's participants prior to the study. For participants who were below 18 years old; parental/guardian consent was obtained, while those 18 years and older signed their own consent. During the implementation phase, the participants were requested to fill up a self-administered questionnaire to provide socio-demographic, economic information and food intake for the previous day.

### 2.2 Dietary Assessment

Dietary diversity was measured using the WHO & FAO one day diversity questionnaire. The diversity questionnaire used consists of 14 groups of foods, which covers almost every food taken. In addition the questionnaire had a single question about any food or fast food consumed out of the house. We evaluated one usual day in the week except holidays. We used the FAO questionnaire form from FANTA Household Dietary Diversity Questionnaire 2006 (expanded version) and the questionnaire used for women in the Demographic and Health Surveys (DHS) for data gathering. Using individual –level questionnaire is suitable for anyone aged more than three years. Dietary diversity scores of food groups is a simple count of the family or individual has consumed during the previous 24 hours (Hoddinott & Yohannes, 2002).

The original questionnaire for dietary diversity was applied after translation to Persian. There was one question for cereals, four questions about vegetables, two about fruits, two about meats, one for eggs, one for fish, one for dairy products, one for legumes, nuts and seeds, and one question for oil and fats. Also, in front of each question, common samples of that group were mentioned. This questionnaire was reviewed by three experts in nutrition, to check the conformity of food items with Iranian foods. Questionnaires were self- administered by students. We used the formula recommended by the FAO for measuring micronutrients in the dietary diversity questionnaire (Hoddinott & Yohannes, 2002).

### 2.3 Anthropometric Measures

Weight and height of participants was measured while their shoes were taken, using a digital electronic scale (SECA 760; range 0.1-150 kg) and a stadiometer (SECA 206, range 0-220 cm). Body Mass Index (BMI) was calculated using the following formula (BMI= weight (kg)/height m2). Overweight and obesity were defined based on the determined BMI cut-off values for adolescents. According to this cut-offs, subjects were divided into four groups: overweight, obese, underweight and normal weight (Must, Dallal, & Dietz, 1991). To measure the waist circumference (WC), the bust's narrowest level was measured and the hip circumference was considered at the maximum level of hip over light clothing. All measurements were taken by one of the researchers to reduce the chances of error.

### 2.4 Analysis

Data entry and analyzing were done using SPSS (Ver. 17) statistical software program. Data were reported as mean ± SD, frequency and percent. The independent t-test was used for comparing two groups (dietary diversity < 6 and ≥6). The chi-square test was used to determine the relationship between the DDS and anthropometric measures. To determining the relationship between the economic situation and DDS, the Kruskal Wallis test was used. Logistic regression was done using DDS as the response variable and evaluating the risk of different determinants on DDS while adjusting for the ethnicity as a confounding variable. Differences were considered significant when *p*< 0.05.

## 3. Results

After excluding the uncompleted questionnaires and those who did not cooperate in the anthropometric measurements, 506 cases (girls) remained in this study for evaluation of dietary diversity. Most of the participants were aged 15-16 (67.7%) and were at the first degree of high school. The socio-demographic characteristics of the participants are listed in Table 1.

**Table 1:** Socio-demographic characteristic of participants

Characteristics	n= 506 Mean ± SD or N(%)
**Age**	15.4 ±1.03
**Type of school**	
Government	465(91.9)
Private	41 (8.1)
**Ethnicity**	
Arab	253 (50)
Fars	146 (28.9)
Other	107 (21.1)
**Grade in high school**	
First	251 (49.6)
Second	135 (26.7)
Third	120 (23.7)
**Economic situation**	
Weak	29 (5.7)
Moderate	177 (35)
Well	261 (51.6)
Well-off	39 (7.7)

The mean and SD of the dietary diversity score was 6.81±1.75. The anthropometric measures of girls are listed in Table 2. As evident in Table 2, 18.85% and 8.3% of participants were overweight and obese respectively. Table 2 shows the correlation between the anthropometric measures, economic situation and dietary diversity score. When distribution of DDS (according to the Food Guide Pyramid) was divided into two groups - less than six and six and over, the Logistic Regression showed that; weak economical situation was a risk for poor DDS (OR= 3.5, 95% CI= 1.06,10.6, p=0.03). There was not any significant difference between DDS<6 and ≥6 regarding age, type of school and ethnicity using the Logistic Regression test.

**Table 2:** Distribution of dietary diversity (DDS) and its relationship with anthropometric measures and economic situation

Characteristics	Dietary Diversity Mean ±SD or N(%)		Test value	P value

	≥6	<6		
**BMI kg/m^2^**	22.1±4.45	22.5±4.4	χ^2^=0.3	P=0.6
<5^th^ percentile	29(85.3)	5(14.7)		
5^th^ – 85^th^ percentile	265(79.1)	70(20.9)		
85^th^ – 95^th^ percentile	73(76.8)	22(23.2)		
>95^th^ percentile	31(73.8)	11(26.2)		
**Waist circumference(cm)**	69.6±8.7	70.7±8.5	χ^2^=0.34	P=0.55
<80	351(79.1)	93(20.9)		
>80	47 (75.8)	15(24.2)		
**WHR**	0.73±0.04	0.74±0.04	χ^2^=3.4	P=0.05
<0.80	382(79.4)	99(20.6)		
>0.80	16(64)	9(36)		
**Economic situation**			χ^2^=9.19	P=0.002
Weak	18 (62.1)	11(37.9)		
Moderate	131(74)	46(26)		
Well	216 (82.8)	45(17.2)		
Well off	33 (84.6)	6(15.4)		

In participants with scores ≥ six, the abnormal BMI, waist circumference and waist hip ratio were non significantly more than individuals with scores of less than six. The micronutrients (vitamin A or iron) were calculated using the formula recommended by the FAO. According to the formula, only 19.98% of participants used vitamin A rich fruits, 7.3% consumed organ meat, 22.13% used eggs, 79.2% consumed dairy products and 16% consumed fish. The questionnaire consisted of a question about fast food consumption. According to this question 102 (20.2%) of the participants ate fast food.

## 4. Discussion

This study showed that the mean DDS was 6.81±1.75. This result is consistent with other studies that measured DDS using the 24- hour recall questionnaire among adolescents (Mirmiran, Azadbaht, Esmailzadeh, & Azizi, 2004; Azadbaht & Esmaillzadeh, 2010).

All anthropometric measures in our study were slightly higher in participants with DDS ≥6. In a study by Mirmiran et al., the result showed that; participants in the DDS≥ 6 were mostly girls and had higher BMI than those in the lower category (19.81 ± 4.07 vs 18.95 ± 3.30 kg/m2, *P*<0.01) (Mirmiran et al., 2004).

Also, in our study, there was significant correlation between the waist hip ratio with dietary diversity. Our results are similar to other studies on youth in Iran (Azadbaht & Esmaillzadeh, 2010).

In the present study, around 100% of girls consumed cereals, 50% consumed green leafy vegetables and more than 50% consumed other vegetables. It seems that the DDS improved when consumption of healthy food groups increased. Higher DDS is not always associated with increased weight gain, because it may due to the increase in consumption of low calorie food such as vegetables, whole grains and fruit (Mirmiran et al., 2004).

The results of the study showed that only 19.98% of participants used vitamin A rich fruits. A study by Kennedy et al., who used a24 -hour recall questionnaire for measuring dietary diversity, showed that the median intake of vitamin A was below the estimated average requirements (Kennedy, Fanou, Seghieri, & Brouwer, 2009). In Mirmiran et al.'s study (2004), the researchers found that; less than 25% of all adolescents had an adequate fruit intake.

The questionnaire included a question about fast food consumption. Around 80% of the participants claimed that they did not consume fast food in the last 24 hours. Studies showed that about 33 percent of children and adolescents in the United States consume fast food in a typical day, and that the intake increases with age (Bowman et al., 2004; Paeratakul et al., 2003).

In our study, the mothers of most students’ were housewives which might have contributed to the lower consumption of fast foods by students. In a study in Isfahan, the results indicated that although the percentage of fat intake was according to the recommended fat intake allowance for adolescents, in most cases, the level of serum total cholesterol, low-density lipoprotein and triglyceride were significantly greater than the standards for age. These results indicate that an improper intake of high amounts of saturated fat and fast food among Iranian adolescents might not be detected by the questionnaire (Kelishadi et al., 2004).

There was a significant relationship between the DDS and economic situation. In a study has done by Labadarios et al. (2011), they found that; there were significant differences in the DDS by the living standards mean (LSM) analysis (p < 0.05) when the decrease in LSM was associated with lowest mean DDS (2.93). Poor people often do not have access to a diverse food. Although access is important, but the awareness of food-based dietary guidelines will probably have more effect. Furthermore, it is evident that food diversity needs greater cost.

This is the first study on the dietary diversity of adolescents in Ahvaz, Iran, with a proportionally large and random sample size. In addition it is the first time that we used the FAO's third version of the guidelines for measuring household and individual dietary diversity. Other studies about dietary diversity in Iran used 24- hour recall for data gathering.

### 4.1 Limitation

The study has limitations. The estimation of the economic situation of families is not simple and or correct, as generally, students and families do not reflect their real economic situation due to the fear of tax and other social matters. The FAO's third version of the guidelines for measuring household and individual dietary diversity only asked about the last 24 hours might not be a correct reflection of the usual habits of adolescents.

## 5. Conclusions

The FAO's third version of the guidelines is a good indicator for measuring the dietary diversity score. The results of this study indicate that high school girls’ knowledge and practice about dietary diversity were not satisfactory and need to be improved by educational classes about dietary diversity.
